# A SNP‐based consensus genetic map for synteny‐based trait targeting in faba bean (*Vicia faba* L.)

**DOI:** 10.1111/pbi.12371

**Published:** 2015-04-10

**Authors:** Anne Webb, Amanda Cottage, Thomas Wood, Khalil Khamassi, Douglas Hobbs, Krystyna Gostkiewicz, Mark White, Hamid Khazaei, Mohamed Ali, Daniel Street, Gérard Duc, Fred L. Stoddard, Fouad Maalouf, Francis C. Ogbonnaya, Wolfgang Link, Jane Thomas, Donal M O'Sullivan

**Affiliations:** ^1^ National Institute of Agricultural BotanyCambridgeUK; ^2^ PGROThornaughPeterboroughUK; ^3^ Department of Agricultural SciencesUniversity of HelsinkiHelsinkiFinland; ^4^ Department of Crop SciencesGeorg‐August‐UniversitätGöttingenGermany; ^5^ LGC Ltd (Genomics Division)HoddesdonHertsUK; ^6^ INRAUMR1347 AgroécologieDijonFrance; ^7^ ICARDATerbolLebanon; ^8^ School of Agriculture, Policy and DevelopmentUniversity of ReadingWhiteknightsUK; ^9^ Present address: Plant science DepartmentUniversity of SaskatchewanAgriculture Building51 Campus DriveS7N 5A8SaskatoonSKCanada; ^10^ Present address: Agronomy DepartmentCollege of AgricultureAssiut UniversityAssiut71526Egypt; ^11^ Present address: Grains Research and Development Corporation (GRDC) Level 4 East Building4 National CircuitBartonACT2600Australia

**Keywords:** legume, faba bean, KASP genotyping, single nucleotide polymorphism, synteny

## Abstract

Faba bean (*Vicia faba* L.) is a globally important nitrogen‐fixing legume, which is widely grown in a diverse range of environments. In this work, we mine and validate a set of 845 SNPs from the aligned transcriptomes of two contrasting inbred lines. Each *V. faba* SNP is assigned by BLAST analysis to a single Medicago orthologue. This set of syntenically anchored polymorphisms were then validated as individual KASP assays, classified according to their informativeness and performance on a panel of 37 inbred lines, and the best performing 757 markers used to genotype six mapping populations. The six resulting linkage maps were merged into a single consensus map on which 687 SNPs were placed on six linkage groups, each presumed to correspond to one of the six *V. faba* chromosomes. This sequence‐based consensus map was used to explore synteny with the most closely related crop species, lentil and the most closely related fully sequenced genome, Medicago. Large tracts of uninterrupted colinearity were found between faba bean and Medicago, making it relatively straightforward to predict gene content and order in mapped genetic interval. As a demonstration of this, we mapped a flower colour gene to a 2‐cM interval of Vf chromosome 2 which was highly colinear with Mt3. The obvious candidate gene from 78 gene models in the collinear Medicago chromosome segment was the previously characterized MtWD40‐1 gene controlling anthocyanin production in Medicago and resequencing of the Vf orthologue showed a putative causative deletion of the entire 5′ end of the gene.

## Introduction

Faba bean is a globally significant grain legume, with 4–5 million tonnes annual production worldwide in recent years (FAO, 2014), providing the predominant affordable dietary source of protein to subsistence farmers and urban populations across North Africa, Horn of Africa (including Ethiopia) and the Middle and Near East as well as parts of China. The use of faba bean is deeply embedded in the cuisine and culture of these countries and the great agronomic and morphological diversity of the crop genepool has permitted it to become adapted to many agroecological settings and different end uses (Cubero, 1974; Duc *et al*., 2010). As a nitrogen‐fixing legume, it has an important role in maintaining soil fertility in rotational systems (Jensen *et al*., 2010; Köpke and Nemecek, 2010). However, faba bean production and consumption are becoming increasingly geographically disjointed, with alarming reductions in output in precisely those countries with rapidly growing populations that depend most heavily on faba beans as a dietary protein source. For example, Egypt's self‐sufficiency in faba bean production fell from 96% to 52% between 1998 and 2009 due to widespread infestation of the Nile Delta region by *Orobanche crenata* Forsk. and the near abandonment of faba bean production in the middle Nile Valley region due to the prevalence of faba bean necrotic virus (Mahmoud Zeid, pers. commun.). Moreover, the almost unchecked spread of parasitic weeds of the genus *Orobanche* has led to abandonment for faba bean growing of entire provinces not only in Egypt but across the entire Mediterranean basin (Maalouf *et al*., 2009). These as well as other production constraints such as drought and extreme temperature sensitivity could potentially be addressed by more effective genomics‐enabled genetic analysis/breeding strategies.

Despite the global significance of the crop and the urgency of researchable challenges it faces, faba bean genetic and genomic resources have only started to be developed in the last few years, due in part at least to its outcrossing habit and large (approx.13 Gb) genome size. Initially, mapping studies have been based on random amplified polymorphic DNA (RAPD) marker technology and delivered small numbers of cross‐specific trait‐linked sequence characterized amplified region (SCAR) markers (Avila *et al*., 2003, 2004; Diaz‐Ruiz *et al*., 2010; Gutierrez *et al*., 2006, 2007). Later, the development of simple sequence repeat (SSR) and single nucleotide polymorphism (SNP) marker sets potentially more amenable to deployment in genetic studies and breeding have been reported (Ellwood *et al*., 2008; Gong *et al*., 2011; Kaur *et al*., 2012; Ma *et al*., 2011; Wang *et al*., 2011; Zeid *et al*., 2009). The recent development of the gene sequence‐based maps of *V. faba* (Cruz‐Izquierdo *et al*., 2012; Ellwood *et al*., 2008; El‐Rodeny *et al*., 2014; Kaur *et al*., 2014a; Satovic *et al*., 2013) has been a big step forward for faba bean genetics. However, so far, gene‐based linkage maps have either utilized multiple low‐throughput marker systems to achieve assignment of most major linkage groups to one of the six chromosomes (Satovic *et al*., 2013) or generated multiple linkage groups per chromosome, mostly unassigned (El‐Rodeny *et al*., 2014; Kaur *et al*., 2014a,b), and therefore, there is not yet a single genotyping platform for *V. faba* which permits genomewide alignment to a comprehensive consensus map providing coverage of all six chromosomes and supports systematic targeting of marker development to regions of interest exploiting synteny with sequenced legumes.

We previously demonstrated conversion of set of mapped cleaved amplified polymorphic sequence (CAPS) and SNAPshot assays to Kompetitive Allele Specific PCR (KASP) format in faba bean (Cottage *et al*., 2012). Here, we significantly increase the SNP coverage of the faba bean genome by discovering, validating and mapping a much larger number of SNP markers, and further pursue the objective of making all useful SNPs readily accessible by opting for the low‐cost, easy‐to‐use, KASP genotyping platform. We find extensive colinearity of the *V. faba* linkage map with the sequenced model legume *M. truncatula* and demonstrate the potential for application of this new syntenically anchored SNP map in trait dissection and breeding via the identification of a candidate gene/causative polymorphism for white‐coloured flowers.

## Results

### Transcriptome resequencing for SNP discovery

Pyrosequencing and *de novo* transcript assembly of RNA from seedling tissues of two contrasting faba bean lines – NV643‐4, a partly inbred line derived from the white flowered, white hilum Polish variety ‘Albus’ and NV648‐1, a derivative of the ICARDA Bean Pure Line ‘BPL10′, a coloured flower, black hilum inbred line – yielded 17 397 and 16 041 contigs >200 bp in length for NV643‐4 and NV648‐1, respectively. From amongst these 5562 and 5534 contigs of NV643‐4 and NV648‐1, respectively, aligned to contigs from the other *V. faba* line as well as to at least one putative gene of *M. truncatula*. These transcript assemblies are available from the GenBank Transcript Sequence Assembly (TSA) repository under the accession names GARZ00000000 (NV643‐4) and GASA00000000 (NV648‐1). The versions described in this paper are the first versions, GARZ01000000 and GASA01000000, respectively. Tables 1 and 2 show some metrics of the sequencing runs and assembly results.

**Table 1:** Statistics for 454 sequencing of *V. faba* ‘Albus’ and ‘BPL10’

Genotype	Tissue	Total reads	Average raw read length
NV643‐4 (Albus)	10‐day‐old seedling	788 918	525
NV648‐1 (BPL10)	10‐day‐old seedling	751 023	527

**Table 2:** Statistics for transcriptome assembly of *V. faba* ‘Albus’ and ‘BPL10’ in gsAssembler, implemented in Newbler v. 2.3 (Roche)

Genotype	Total contigs	N50	Total coverage %	Contigs in TSA submission	GenBank TSA accession
NV634‐4	26 709	1152	90.10	17 397	GARZ00000000
NV648‐1	21 591	1119	89.72	16 041	GASA00000000

### Information content of validated KASP SNP assays

Of 1001 selected target sequences (data not shown) containing putative NV643‐4/NV648‐1 SNPs, assays were successfully designed for 845 SNPs (for details of individual target sequences, Table S1). To provide continuity with previous work (Cottage *et al*., 2012) and a link to the previously published Vf6 × Vf27 maps (Cruz‐Izquierdo *et al*., 2012; Ellwood *et al*., 2008), the 48 most informative SNP assays developed from the Vf6 × Vf27 map (Cottage *et al*., 2012) were included in the subsequent analyses. With the exception of the aforementioned set of 48 previously described SNPs, all SNPs described here were discovered by alignment of sequences from just a single pair of genotypes. Therefore, we wished to assess allele frequencies in a diverse germplasm sample so that assays could be scored for information content and to quantify any ascertainment bias towards rare alleles, peculiar to one or other of the ‘discovery’ genotypes. A second, overlapping objective was to screen a set of parents of mapping populations that already existed or are under development, so that users of these populations could quickly extend coverage of their linkage maps to encompass polymorphic SNPs from the new set. To fulfil these complementary purposes, a set of 37 inbred lines (the ‘validation panel’) was assembled and genotyped (Table 3). A total of 891 SNP assays were validated and assigned to one of four quality classes based on call rate and tightness of clustering of the three possible genotypic classes. Figure S1 shows raw genotyping data for two SNPs assigned to quality classes I and IV, respectively. For the remainder of analyses, only the 757 SNPs, henceforth referred to as ‘NV648/NV643′ SNPs, belonging to quality classes I and II are considered further. Allele calls for only 67.6% 512 of 757 of the high‐quality ‘NV648/NV643′ SNP assays matched the SNP allele predicted from sequencing (Table S1). Of the 245 mismatched allele calls, 223 were associated with NV643, where unfortunately, individuals from distinct sublines of this accession had been used for transcriptome sequencing and crossing, respectively. For 241 of 245 of the class I‐II SNPs where discovery and genotyped calls were discordant, both alleles were present in the validation panel despite the lack of the predicted NV643‐3 (discovery sequence) allele in NV643‐4 (genotyped) DNA, and therefore, we attribute the majority of mismatches in predicted versus observed allele calls to NV643 stock heterogeneity rather than sequencing errors or flaws in the assay design. Amongst the 512 class I‐II SNPs where discovery sequence and SNP genotype were concordant, 438 were nonprivate with respect to the discovery genotypes as both alleles were observed amongst the 35 nondiscovery genotypes, but amongst the remaining 73 SNPs, the clear majority (60) of minor alleles were private to the NV648‐1 discovery genotype, indicating that this line is the more ‘exotic’ of the discovery genotypes with respect to the diversity sampled in the validation panel. Figure S2 shows the genetic distance relationships between lines of the validation panel. As expected, BPL10 is on the longest branch, reflecting the ascertainment bias of the SNP discovery. However, polymorphism information content (PIC) values were high with an average of 0.316 amongst the 656 nonprivate SNPs. To add SNPs discovered using different discovery genotypes and markers which could be polymorphic both in the previously reported Vf6 × Vf27 gene‐based linkage map and maps created using the new SNPs described here, 40 of the markers from the Vf6 × Vf27 map (Cottage *et al*., 2012), henceforth referred to as ‘Vf6/Vf27′ SNPs were genotyped on the validation panel. The average PIC of this subset of SNPs was 0.329. Heterozygosity amongst the inbred lines using the combined set of 837 class I–II SNPs averaged 1.35%, consistent with the status of these as relatively pure research lines used for genetic analyses.

**Table 3:** Inbred lines of *V. faba*, used to validate panel of SNP markers. The original accessions were provided by National Institute of Agricultural Botany (NIAB), United Kingdom, The Georg‐August‐University, Göttingen (GAUG), Germany; the International Center for Agricultural Research in Dry Areas (ICARDA), Syria; the Instituto de Investigación y Formación Agroalimentaria (IFAPA), Spain; the University of Helsinki (UoH) Finland; and the Institut National de la Récherche Agronomique (INRA), France

Accession code	Other reference	Donor	% Homo‐zygosity	% Hetero‐zygosity
NV153‐1	ig12658	NIAB	98.73	1.27
NV639‐1	Hedin	GAUG	98.61	1.39
NV643‐3	Albus	NIAB	97.85	2.15
NV644‐1	Kasztelan	NIAB	92.56	7.44
NV648‐2	BPL10	ICARDA	98.87	1.13
NV656‐3	ig101942	NIAB	99.37	0.63
NV657‐2	INRA‐29H	IFAPA	99.11	0.89
NV658‐2	CGN07715 cf‐3	GAUG	99.24	0.76
NV662‐1	VF136	IFAPA	97.22	2.78
NV713‐1	Côte d'Or	GAUG	98.36	1.64
NV714‐1	Hiverna	GAUG	99.25	0.75
NV715‐1	Webo	GAUG	99.10	0.90
NV716‐1	Wibo	GAUG	99.36	0.64
NV717‐1	L79‐79	GAUG	99.50	0.50
NV718‐1	L977‐88	GAUG	99.75	0.25
NV719‐1	L979‐S1	GAUG	99.62	0.38
NV720‐1	Bourdon	GAUG	99.24	0.76
NV721‐1	Arrisot	GAUG	98.99	1.01
NV722‐1	Banner	GAUG	98.99	1.01
NV723‐1	Bulldog	GAUG	99.12	0.88
NV724‐1	Pietranera	GAUG	99.24	0.76
NV725‐1	GIZA3‐2	GAUG	99.36	0.64
NV726‐1	GIZA402	GAUG	99.11	0.89
NV727‐1	BPL4628	ICARDA	98.86	1.14
NV728‐1	TW	ICARDA	99.11	0.89
NV729‐1	VF6	ICARDA	98.58	1.42
NV730‐1	ILB4347‐4	ICARDA	99.24	0.76
NV731‐1	ILB4347‐3	ICARDA	99.50	0.50
NV732‐1	BPL710	ICARDA	97.83	2.17
NV733‐1	NA112	ICARDA	99.12	0.88
NV734‐1	ILB938	UoH	99.62	0.38
NV735‐1	Mélodie	UoH	98.99	1.01
NV736‐1	Aurora	UoH	98.22	1.78
NV737‐1	CRB285	INRA	97.11	2.89
NV738‐1	CRB2516	INRA	99.21	0.79
NV739‐1	CRB2702	INRA	94.63	5.37
NV740‐1	CRB100107	INRA	99.60	0.40

### Towards a consensus SNP‐based genetic linkage map

**Figure 1:**
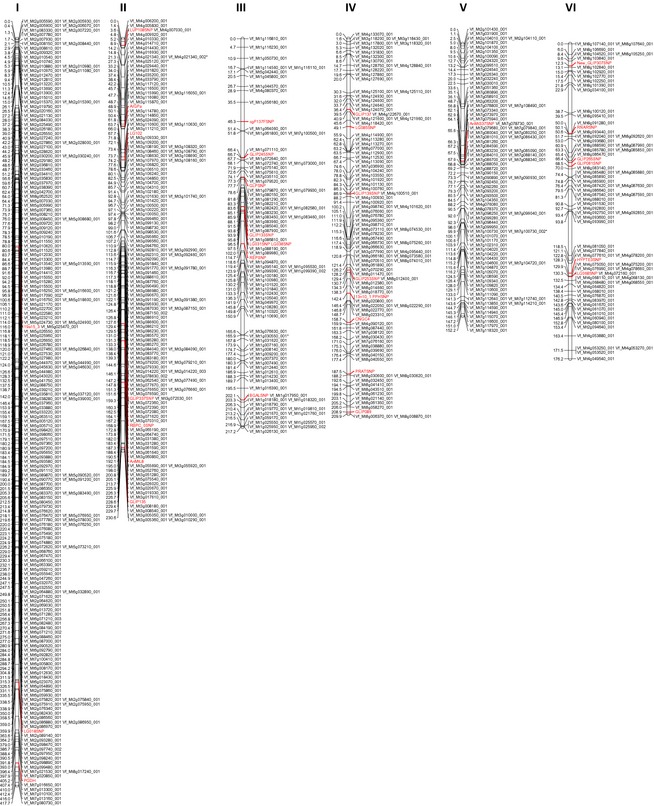
A 687‐locus consensus SNP‐based map showing the six linkage groups thought to represent the six physical chromosomes of *Vicia faba*. Markers in red have been developed in previous studies. Graph created using MapChart (Voorrips, 2002).

The six mapping populations listed in Table 4 were used to construct first individual linkage maps and in a second step, a consensus map for *V. faba*. The framework for the consensus map was provided by the NV648‐1 × NV643‐4 F_2_ linkage map, where 470 of the 757 ‘NV648/NV643′ and 10 of the ‘Vf6/Vf27′ SNP markers could be placed in just six linkage groups. The other five maps were more fragmented with between 12 and 20 linkage groups and less well populated with between 109 and 231 SNPs. Nonetheless, 207 SNPs not placed in the framework NV648‐1 × NV643‐4 F_2_ map were added to the consensus map through the integration of these additional populations. The final consensus map constructed using Merge Map (Wu *et al*., 2011), referred to as the Vf 2014 Consensus, consisted of 687 SNPs (653 ‘NV646/NV643′ and 34 ‘Vf6/Vf27′ SNPs) corresponding to 542 unique, that is non‐co‐segregating, loci on six linkage groups and spanning a total length of 1403.8 cM (Figure 1, Table S2). The average gap size in this map is 2.6 cM, and the largest single gap is 24.7 cM. We postulate that these six linkage groups correspond to the six physical chromosomes. Based on the distribution of ‘Vf6/Vf27′ SNPs from linkage groups previously assigned to physical chromosomes (Cruz‐Izquierdo *et al*., 2012; Ruiz‐Rodriguez *et al*., 2014), we have numbered these six linkage groups I‐VI to reflect the physical assignments already made (Table S3).

**Table 4:** Mapping populations genotyped with SNP markers developed in this study

Population	Male parent	Female parent	progeny analysed	mapped SNPs	linkage groups	map length (cM)	Segregating traits
1	NV643‐1	NV648‐1	136 F2	481	6	1166.5	Flower colour (white/wild type)
2	NV643‐1	NV657	165 F2	231	13	983.3	Flower colour (white/wild type)
3	NV639	NV658	52 F2	204	18	1198.9	Flower opening (closed/open)
4	NV644‐1	NV153‐1	125 F2	192	12	1164.9	Plant height (dwarf/tall)
5	Mélodie/2	ILB938/2	194 F5	200	13	818.2	Vicine‐convicine (high/low), stipule spot (present/absent)
6	Côte D'Or/1	BPL4628/1521	101 RILs	108	20	158.1	Frost hardiness

### Colinearity of the faba bean genetic map with the sequenced *M**. **truncatula* genome and the first SNP map of *Lens culinaris*

Synteny between the ‘Vf 2014 Consensus’ linkage map and the *M. truncatula* Mt3.5 pseudomolecule assembly, and the first comprehensive SNP map for lentil (Sharpe *et al*., 2013) and *M. truncatula* was investigated by assigning orthologous links to reciprocal best BLAST hits between the Medicago genome, SNP‐flanking sequence from *V faba* and SNP‐containing contigs assembled from several lines of lentil. The comparative map view shown in Figure S3 represents the full set of links thus obtained at a BLAST cut‐off of E^−30^ and portrays a relatively simple pattern of colinearity between the respective genomes. Mt1 is colinear in a single, virtually uninterrupted block with Vf Chr III and in lentil to the central part of Linkage group 1 (LC_1) which is thought to represent chromosome 1 of *L. culinaris*. Similarly, Mt2 appears colinear to Chr 1 in bean and Lc2 in lentil. Also, Mt3 and Mt6 chromosomes appear colinear to substantial blocks of Vf Chr II and I in faba bean and Lc3 and Lc2 in lentil, respectively. Mt7 is colinear to blocks of Vf Chr V and I in faba bean and Lc 8in lentil, and Mt8 is colinear with large blocks in Vf Chr VI and Chr IV in bean and smaller areas of Lc1, 4 and 7 in lentil. Large parts of Mt4 are syntenous to Vf Chr II,IV and VI and Lc4 and Lc7. This broad picture of high degree of conservation of gene order over few, extensive blocks of colinearity suggests that the gene content and order of *V. faba* genetic intervals currently lacking SNP coverage can be relatively straightforwardly and accurately predicted from aligned stretches of the *M. truncatula* reference genome sequence.

### Mapping of the *ZT1* zero‐tannin end‐use quality trait

Condensed tannins have long been recognized as antinutritional factors when faba bean is used as the main protein source in feed formulations for monogastric livestock and poultry (Bond, 1976). Previous work has shown that the absence of the precursors of condensed tannins, the anthocyanins, in floral tissues, resulting in a highly recognizable white flower phenotype, is pleiotropic to the absence of condensed tannins in the seed coat (Bond, 1976; Picard, 1976). The zero‐tannin (*zt*) trait as it is called, and has been genetically defined as a simple character, governed independently by two complementary recessive genes *zt‐1* and *zt‐2* (Cabrera and Martin, 1989; Picard, 1976). SCAR markers linked to the more common *zt‐1* source of zero tannin/white flower were identified by bulked segregant analysis (Gutierrez *et al*., 2007) but the resulting linkage group was not associated with any particular chromosome and the sequence of the markers gives no clue as to their location in the *V. faba* genome. The segregation of the white flower phenotype in the NV648 (♀) × NV643 (♂) F_2_ population showed a simple 3 : 1 coloured‐to‐white ratio, indicating a single dominant gene was responsible, with the dominant allele coming from the coloured parent. The trait mapped to Vf chromosome 2, in a region of highly conserved synteny with Medicago chromosome 3, between markers Vf_Mt3g092810_001 and Vf_Mt3g094760_001 (Figure S4A and B).

### Use of synteny to target marker development to regions of interest and identify candidate genes

The Medicago chromosome segment lying between flanking markers Vf_Mt3g092810_001 and Vf_Mt3g094760_001 corresponds to approx. 0.57 Mb of DNA with 78 predicted gene models (Figure S4B). Most of these encode hypothetical proteins associated with various retroelements and just two of these were considered as positional candidates for regulating the expression of flower colour in *V. faba*.

A gene ortholog of the Medicago WD‐40 transcription factors, Transparent Testa Glabra 1 (*TTG1*) (Medtr3g092830, Medtr3g092840), was identified in the interval between Vf_Mt3g92810_001 and Vf_Mt3g094760_001. Roles in master regulation of the anthocyanin biosynthesis pathway for *TTG1* orthologs have been suggested in both *Arabidopsis thaliana* (Walker *et al*., 1999) and *M. truncatula* (Pang *et al*., 2009), whereas a deletion in the *A2* gene, an ortholog of Medtr3g092840 in *Pisum sativum*, has been demonstrated to be responsible for a loss of flower colour (Hellens *et al*., 2010). Their known conserved role in regulation of flower and seed anthocyanin production across Arabidopsis, *Medicago* and pea therefore made these compelling biological candidates for regulating anthocyanin production and thus flower pigmentation in *V. faba*. We therefore proceeded to investigate whether sequence variation at the Vf_Mt3g092840 locus, which we will henceforth refer to as *VfTTG1*, could be responsible for the loss of flower colour in NV643. The coding region of *VfTTG1* was cloned and sequenced from NV648 (Figure S4C). However, the 5′ end of the *VfTTG1* sequence could not be amplified in the white‐flowered NV643 line, suggesting a sequence difference or deletion affecting the 5′ priming site. Genome walking was utilized to obtain the sequence of the 5′ region of the *VfTTG1* locus in NV643. This showed that the entire 5′ end of *VfTTG1* gene and upstream promoter elements were missing. The 5′ flanking sequence instead showing high levels of homology to the *VfENOD11* gene (a 213 bp region) and an adjacent region of 388 bp with homology to the *PsIPT1* locus, encoding adenylate isopentenyltransferase) as illustrated in Figure S4D. Primers designed to amplify across the putative deletion amplified a fragment of the expected size (data not shown). An allelism test conducted by crossing NV643 to known sources of *zt‐1* and *zt‐2* allowed us to conclude that *VfTTG1* is in fact, *ZT1* (data not shown). Taken together, the allelic variation, positional and functional data from related taxa strongly indicate that a deletion in the coding region of *VfTTG1*/*ZT1* in *V. faba* is responsible for the loss of floral pigmentation in NV643 and demonstrates how translational functional genomics exploiting well‐described synteny between *Medicago* and *V. faba* can be used to quickly and accurately identify causative genes contributing to specific phenotypes in a crop.

## Discussion

### Emergence of *V. faba* from ‘orphan legume’ status

In the space of just over 1 year, faba bean entries in GenBank have gone from 5000 EST reads (Ray and Georges, 2010) to multiple transcriptomes (Kaur *et al*., 2014a,b; this study). Like pigeon pea, groundnut, lentil and other legumes, there is now a linkage map available with a density of marker coverage appropriate to most currently available RIL mapping populations.

Legume crop species now enjoying low‐medium density SNP coverage of their genomes include cowpea (Muchero *et al*., 2009), common bean (Cortés *et al*., 2011), chickpea (Hiremath *et al*., 2012), lentil (Sharpe *et al*., 2013), pea (Deulvot *et al*., 2010) and faba bean (this work).

### Uses and usability of *Vicia faba* SNP markers in marker‐assisted breeding

Exploitation of molecular markers depends on many factors: their information content, entry cost, per data point cost, infrastructural requirements, ability to access information and coherence as a set of markers likely to provide reasonable genome coverage. We consider that we have attempted to address every single one of these bottlenecks. Our data show that PIC values are high, presumably as a consequence of high outcrossing rate, which suggests that these markers will be broadly useable in each of the main recognized seed size based gene pools (‘major’, ‘equina’ and ‘minor’). The KASP competitive PCR fluorescence assay chemistry was chosen with two particular criteria in mind: firstly, minimizing the cost to researchers of conducting low‐ to medium‐resolution QTL scans and secondly, to cater for marker‐assisted selection in real‐world breeding programmes, where the cost per progeny eliminated on the basis of marker genotypes must be just pennies, ruling out any currently available genomewide genotyping technologies. Many mapping populations are available in the community and we hope that the lower cost barriers will make it possible for more groups to be involved in trait mapping than are currently. For example, SNP markers from this study found to be polymorphic in population 5 (Table 4) have been used to map QTL for stomatal traits (Khazaei *et al*., 2014), low vicine–convicine (Khamassi *et al*., 2013; Khazaei *et al*., 2015) and a novel anthocyanin pigmentation locus, *ssp1* (Khazaei *et al*., 2014b). Similarly, SNP markers from this study used to generate the linkage map for population 4 (Table 4) have been found linked to a novel dwarfing locus, *Dwf1* (K. Khamassi, K. Gostkiewicz, and D.M. O'Sullivan, Unpublished data) and associated with frost tolerance (Sallam, 2014). Furthermore, to facilitate redesign of individual markers to suit existing laboratory set‐ups and locations not easily served by KASP chemistry and commercial genotyping services, an interactive database‐driven website (O'Sullivan and Green, 2012) has been set up to allow users to click from a marker name/location through to a choice of viewing or downloading the original sequence alignment on which the SNP assay design was based as well as accessing clustering profiles, genotype scores and links to any associated references.

These data can be used to pick informative sets of SNPs discovered from four diverse genetic backgrounds and used to efficiently capture genomewide genetic relationships, measure diversity, map traits and once mapped, use marker‐assisted selection schemes to breed for those traits.

### Synteny‐based mapping

Our results broadly support, but refine and extend the conclusions of Cruz‐Izquierdo *et al*. (2012) and Kaur *et al*. (2014a) regarding synteny between the eight *M. truncatula* chromosomes and the six *V. faba* chromosomes. The simple pattern of few, large blocks of colinearity between *V. faba* and *M. truncatula* reported here therefore greatly simplifies the task of translational functional genomics.

Synteny‐based candidate gene discovery as practised for example in cowpea (Muchero *et al*., 2009) is now becoming a reality in faba bean. The identification of a candidate for the zero‐tannin trait illustrates this very well as the SNP markers most closely linked to the zero‐tannin locus occur within a large block of colinearity between Mt3 and Vf LG2 in which a plausible candidate gene is found.

### Towards isolation of causative genes for important trait variation

Tannins (condensed polyphenols) are considered antinutritional compounds limiting the inclusion rates of faba bean in some animal feed formulations. It has been established for a long time that tannins are absent from the testae of white‐flowered bean lines, both phenotypes being manifestations of the absence of anthocyanin production. Recessive alleles at two separate complementary loci (*zt1* and *zt2*) act independently to abolish anthocyanin production, testa tannins and flower colour markings. RAPD‐based SCAR markers linked to *zt1* were previously isolated using a bulked segregant analysis approach although these SCARs were not diagnostic for white flower colour in a panel of 37 European inbred lines and the linkage groups produced in this study did not contain any markers appearing on subsequent *Vicia faba* linkage maps (Cruz‐Izquierdo *et al*., 2012). Our results show that when the zero‐tannin trait was placed on this syntenically anchored map, a rapid inspection of annotated gene content of the colinear region of the Mt3.5 genome revealed tandem copies of the WD40‐repeat containing transcription factor, whose *Arabidopsis* orthologue had been functionally characterized as the *TRANSPARENT TESTA GLABRA1* gene (Pang *et al*., 2009; Qi *et al*., 2011; Sagasser *et al*., 2002; Walker *et al*., 1999; Zhao *et al*., 2008). Although a causative polymorphism controlling determinate flowering trait in faba bean was previously isolated by amplification of the presumed functional ortholog of the *TFL1* gene (Avila *et al*., 2007), this is, to our knowledge, the first time in faba bean that it has been possible to pinpoint a plausible causal polymorphism by combining a syntenically anchored SNP consensus map and sequence and gene function information from Medicago. The syntenic framework presented here provides a basis for the reconstruction of predicted Vf gene order encompassing the large majority of genes that fall within genetic intervals of nearly uninterrupted synteny. A ‘genome zipper’ of this nature is an essential stepping stone to exploit the power of population‐based and bulk segregant mapping‐by‐sequencing approaches (Gardiner *et al*., 2014).

## Experimental procedures

### Plant material

The faba bean inbred lines used in this study either for transcriptome resequencing or SNP validation and their sources are listed in Table 3, as well as all mapping population parents. Genetic linkage mapping was conducted using six biparental populations, further details of which are presented in Table 4.

### RNA library preparation and sequencing

RNA was extracted from complete 7‐day‐old seedlings of part inbred line NV643‐4 (Albus) and inbred line NV648‐1 (BPL10) using the Qiagen RNeasy Plant Mini Kit, and sequenced using Roche 454 GS‐FLX Titanium chemistry. Raw reads were assembled in gsAssembler, based on Newbler v2.3, available free from Roche Life Sciences at http://454.com/products/analysis-software/index.asp (Anonymous, 2010).

CDS, whole transcript and protein sequences from *M. truncatula* were obtained online from the *M. truncatula* Genome Sequencing Project, led by the International Medicago Genome Annotation Group (IMGAG). Version Mt3.5 (Data freeze: 12/31/2009, Assembly release date: 02/10/2010, annotation release date: 05/27/2010) of the annotated genome of *M. truncatula*, consisting of 8 chromosomes and some unanchored BACs was downloaded from http://www.medicagohapmap.org/downloads.php on 01 December 2010.

### SNP discovery and validation

Assembled contigs from NV643‐4 and NV648‐1 were aligned to each other and to transcript sequences of *M. truncatula* using BLASTn. All contigs which gave unique reciprocal best hits over at least 100 bases at a BLASTn cut‐off of E^−30^ in the respective faba bean transcriptome assemblies and to a single predicted *M. truncatula* gene over more than 100 bases were selected for further analysis.

The complete contigs of each NV643‐4:NV648‐1 pair were aligned using ClustalW (Chenna *et al*., 2003) and alignments containing SNPs more than 50 bp from either end of a contig were flagged for marker development. For each SNP, a consensus sequence with the target SNP and at least 50 bp either side of it was then searched against the database of *M. truncatula* pseudomolecules v.3.5, using the BLASTn function (http://www.medicagohapmap.org/advanced_search_page.php?seq). Any consensus which matched multiple chromosomes of *M. truncatula* or in multiple copies on the same chromosome or whose *M. truncatula* alignments contained introns located closer than 50 bp of either side of the SNP were excluded. Sequences with more than 50 bp between the target SNP and the nearest intron site on the corresponding *M. truncatula* contig were truncated at the nearest intron–exon junctions to avoid primers being designed across intron–exon splice sites which would not anneal to genomic (intron‐containing) sequence. The remaining SNP‐containing sequences were then searched in BLASTn against all contigs of NV643‐4 and NV648‐1. Any SNP‐containing alignments with hits to more than one contig from each line were excluded. A total of 887 KASP probes were designed by LGC Genomics Ltd. and each assay given an internal LGC Genomics ID as well as an assay name following the format Vf_MtXgYYYYYY_ZZZ where X indicates the Medicago chromosome number, YYYYYYY the predicted gene identifier of the reciprocal best BLAST hit *M. truncatula* orthologue as given in version Mt3.5 of the Medicago genome assembly (Young *et al*., 2011), and ZZZ the number of the assay (Table S1).

A panel of 37 lines of *V. faba* (Table 3) chosen to represent the parents/founders of key mapping population resources was assembled and DNA extracted from a seedling leaf of a single plant per line using a modified Tanksley method (Fulton *et al*., 1995). Designed KASP assays were genotyped across this panel according to the manufacturer's instructions.

### SNP‐marker quality filtering

Clustering of alternate homozygous SNP alleles and heterozygous genotypes was manually inspected using LGC Genomics SNP viewer. Each SNP assay was scored on a I–IV scale according to tightness of clustering and the number of missing data calls. As an additional criterion on which to rank assays, gene diversity, often referred to as expected heterozygosity, a measure of how informative individual assays are, was calculated as 2pq where *p* is the frequency of the first allele and *q* (the frequency of the alternate allele). Lines or markers with more than 6% no‐calls were excluded in an iterative process. Markers were regarded as very skewed and removed, if either one of the homozygous parental alleles was absent or the homozygous to heterozygous ratio was >2.1 or <0.4 for F2 populations 1–4 and >0.25 or <0.045 for populations 5 and 6, respectively (Table 4).

### Linkage mapping

MapDisto 1.7.5.1 for Windows (Excel 2007 version) was used to find and refine linkage groups (Lorieux, 2012). The LOD was set to five and the map was generated using the procedure Automap. For calculation of all individual linkage maps, the maximum permitted recombination fraction, the parameter *r* in MapDisto, was set to 0.3, except for population four where the use of a more stringent setting of 0.2 was required to avoid spurious linkage. The consensus map was generated in MergeMap (Wu *et al*., 2008) from the linkage groups generated in MapDisto. Graphs were produced using MapChart (Voorrips, 2002).

### Synteny analysis

The flanking sequences of all mapped SNP markers and all lentil contigs were searched in BLASTn against *M. truncatula* CDS version Mt3.5 with an E‐value cut‐off E^−37^. The results were displayed in Strudel (Bayer *et al*., 2011). Discontinuous megablast was used to detect sequence homologies in the flanking region of the recessive VfWD40‐1 allele from NV643.

### Trait targeting by synteny

The coding sequence of Vf_Mt3g092840 (*VfTTG1*) was amplified using standard PCR from NV648 using oligonucleotides: VfTTG1.F1: 5′‐ATGAGATCTAAAACTACGCCTGTGG/VfTTG1.R1: 5′‐TCAAACTCGAACCCTCAAAAGC (0.5 μm) using Phusion taq polymerase (N.E.B., Hitchin, UK). Thermal cycling conditions: 96 °C for 5 min, (30 × 96 °C/15 s, 58 °C/15 s, 72 °C/30 s) and 72 °C/10 min final extension. Products were cloned into a pGEM‐T Easy vector (Promega, Southampton, UK) and sequenced using BigDye V3.0 (Life Technologies, Paisley, UK).

A genome walking kit (Clontech, Mountain View (CA), USA) was used in conjunction with the VfTTG1.R1 primer to generate primary amplification products for walking. A nested primer (VfTTG1.N) 5′‐TCAAACCCTCAAAAGCTGCATCTTGTTAG was then used for walking upstream generating a 982‐bp product. This was cloned and sequenced as described previously.

## Supporting information


**Figure S1** Examples of SNP calls for the SNP validation panel of 37 inbred *V. faba* lines (Table 3) displayed in SNPViewer (LGC Ltd., UK) for a marker assigned to quality class I (top) and IV (bottom).
**Figure S2** UPGMA dendrogram of the SNP‐validation panel of 37 inbred *V. faba* lines, based on the panel of SNP markers developed in this study.
**Figure S3** Display of synteny between chromosomes of *M. truncatula* (centre) and linkage groups likely to represent chromosomes of *V. faba* (left) and *L. culinaris* (Sharpe *et al*., 2013) (right).
**Figure S4** A. A portion of the linkage map from the F_2_ population 1 (NV643‐1 × NV648‐1) showing the map location of ZT1 between flanking markers Mt3g094760_001 and Vf_Mt3g092810_001.

**Table S1** SNPs identified in *V. faba* lines Albus and BPL10, their flanking sequences and alleles, quality score and PIC values.
**Table S2** Consensus linkage map created in MergeMap from from separate individual maps listed in Table S4.
**Table S3** Markers mapped here and in previous studies and their assignment to linkage groups of three maps created for *V. faba*.
**Table S4** Linkage groups from individual maps used to produce the consensus map in this study. Individual maps from populations listed in Table 2 were produced in MapDisto and given a weighting factor.
