# Supplementary material for: A SNP‐based consensus genetic map for synteny‐based trait targeting in faba bean (Vicia faba L.)
Source: Plant Biotechnol J. 2015 Apr 10;14(1):177–85. doi: 10.1111/pbi.12371 (PMC4973813; doi:10.1111/pbi.12371)
Supplement: Supplementary file 1 — Figure S1 Examples of SNP calls for the SNP validation panel of 37 inbred V. faba lines (Table 3) displayed in SNPViewer (LGC Ltd., UK) for a marker assigned to quality class I (top) and IV (bottom). Figure S2 UPGMA dendrogram of the SNP‐validation panel of 37 inbred V. faba lines, based on the panel of SNP markers developed in this study. Figure S3 Display of synteny between chromosomes of M. truncatula (centre) and linkage groups likely to represent chromosomes of V. faba (left) and L. culinaris (Sharpe et al., 2013) (right). Figure S4 A. A portion of the linkage map from the F2 population 1 (NV643‐1 × NV648‐1) showing the map location of ZT1 between flanking markers Mt3g094760_001 and Vf_Mt3g092810_001. [file PBI-14-177-s001.pdf]

Supplementary Figures

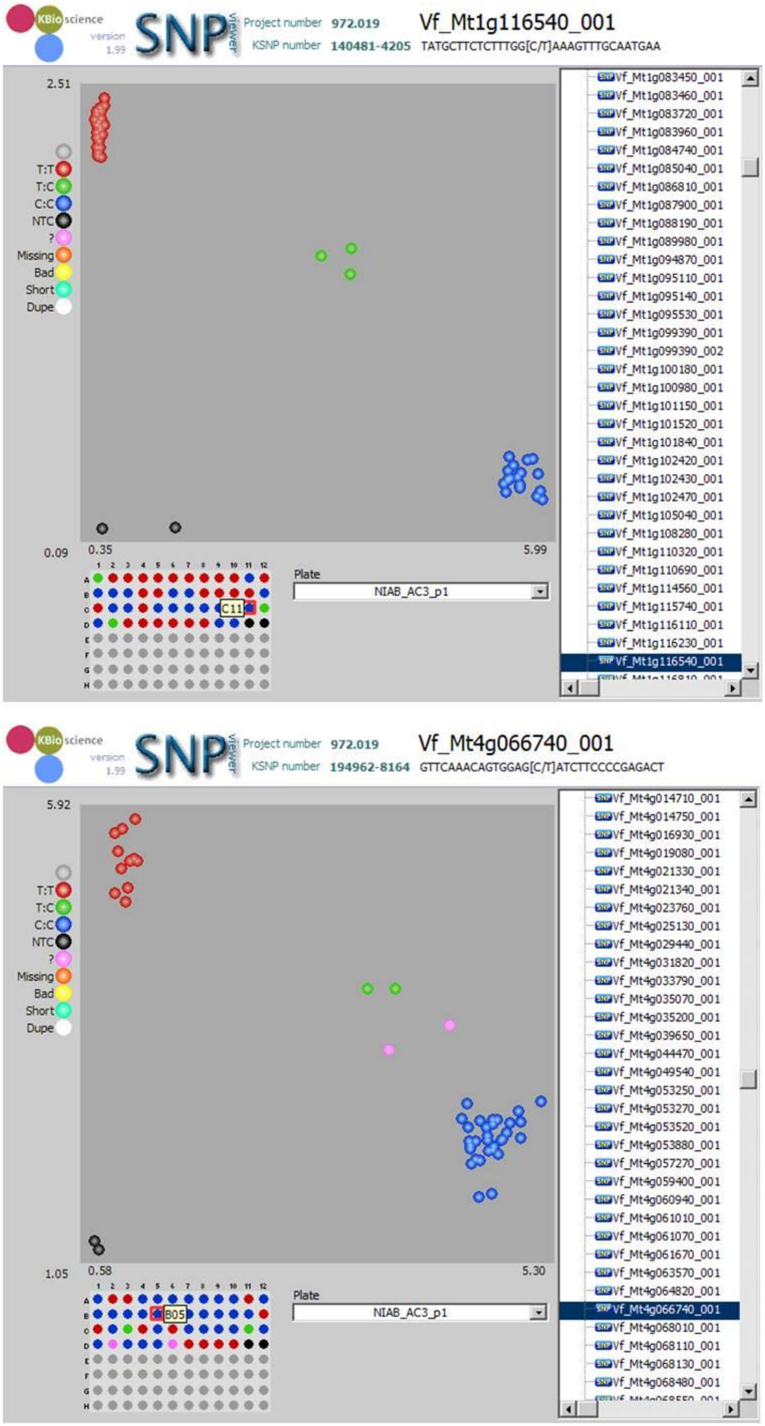

Supplementary Figure 1: Examples of SNP calls for the SNP validation panel of 37 inbred *Vicia faba* lines (Table 3) displayed in SNPViewer (LGC Genomics Ltd., UK) for a marker assigned to quality class I (top) and IV (bottom).

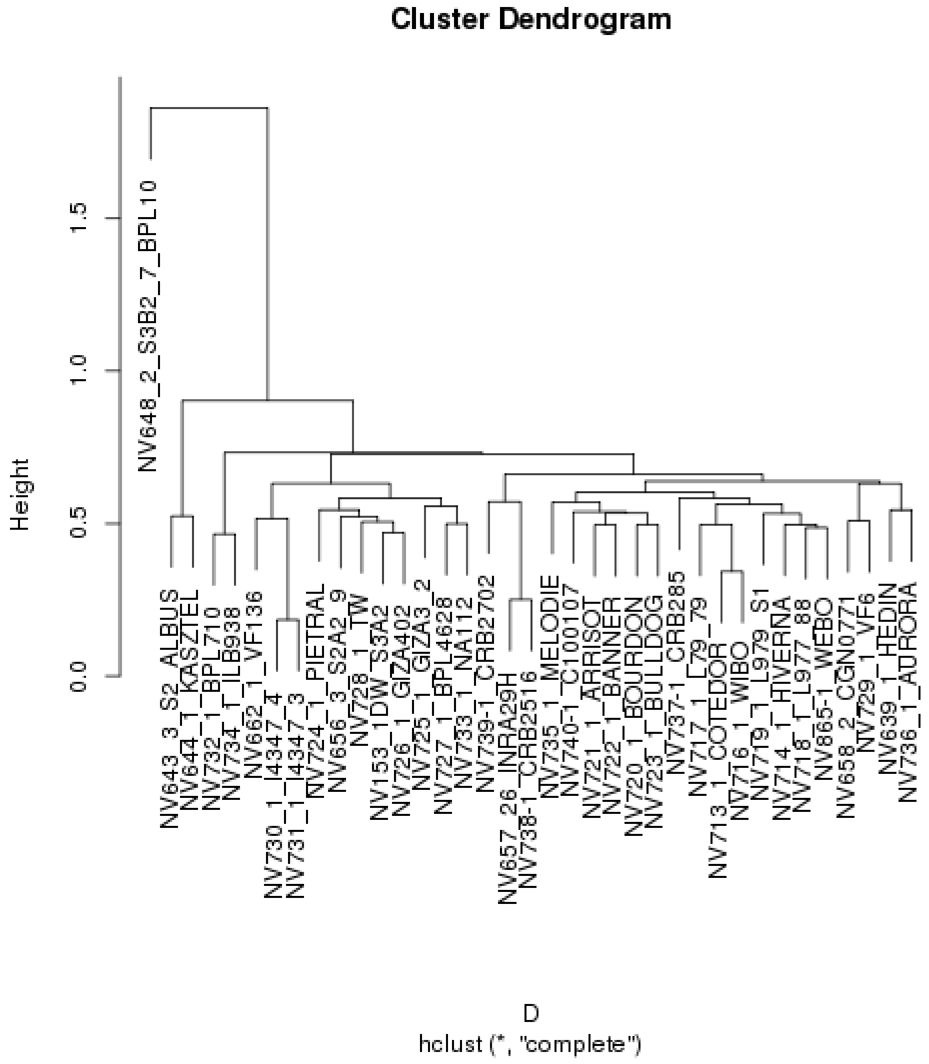

Supplementary Figure 2: UPGMA dendrogram of the SNP-validation panel of 37 inbred *V. faba* lines , based on the panel of SNP markers developed in this study. Line identifiers shown here are a concatenation of the NIAB Vicia (NV) accession number and given name of the inbred shown in Table 3.

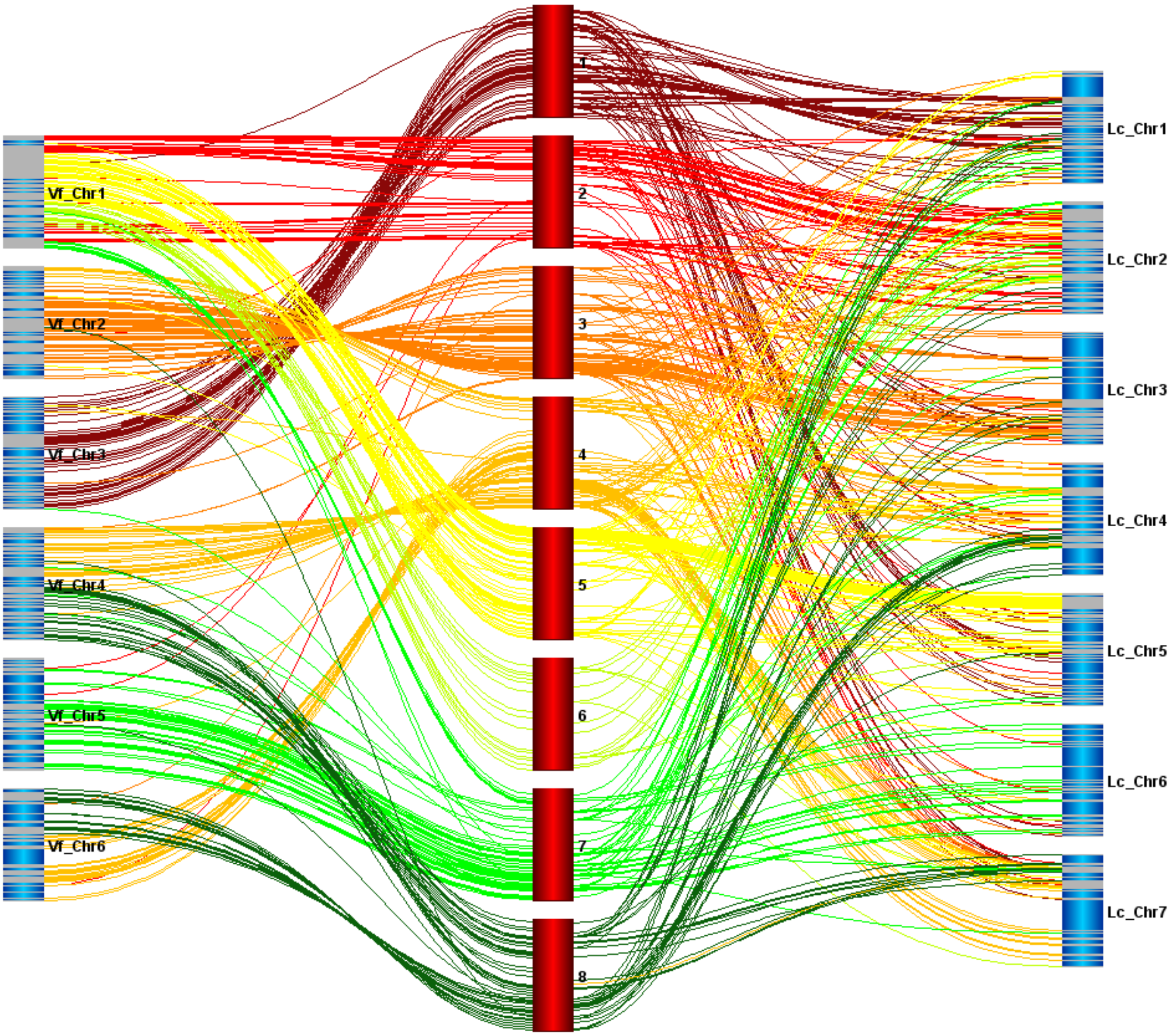

Supplementary Figure 3: Display of synteny between chromosomes of *Medicago truncatula* (centre) and linkage groups likely to represent chromosomes of *Vicia faba* (left) and *Lens culinaris* (Sharpe et al. 2013) (right). For *Vicia faba,* only markers syntenous to one locus on *M. truncatula* were mapped, markers of *L. culinaris* may map to more than one locus in *M. truncatula*.

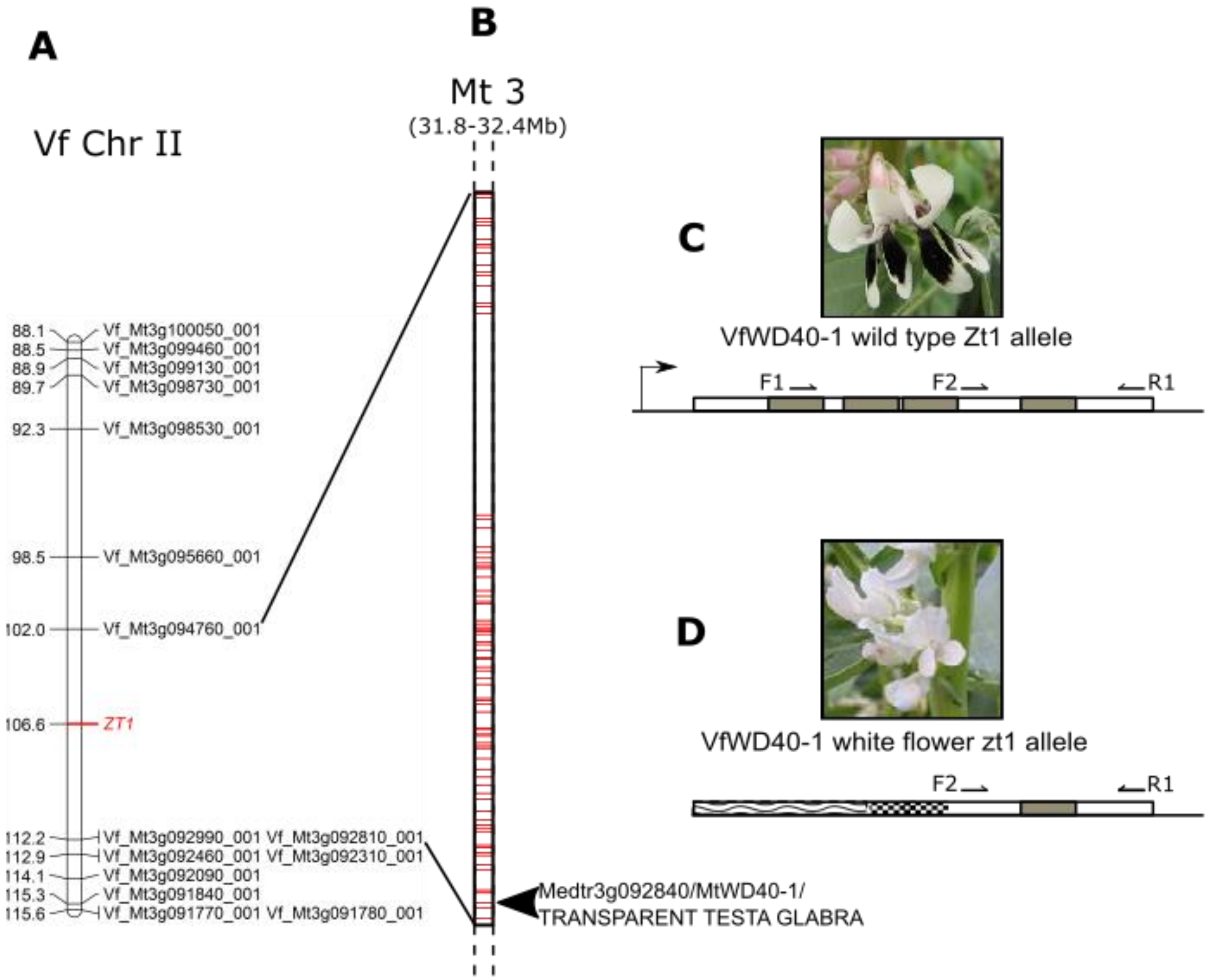

Supplementary Figure 4: **A.** A portion of the linkage map from the F_2 population 1 (NV643-1 x NV648-1) showing the map location of ZT1 between flanking markers Mt3g094760_001 and Vf_Mt3g092810_001. **B.** A schematic of the collinear region of M. truncatula showing the locations of the 78 predicted genes between Medtr3g098210 and Medtr3g09470 (horizontal red ticks). The most obvious candidate functional orthologue for *ZT1* in this interval is Medtr3g092840, annotated as TRANSPARENT TESTA GLABRA and functionally characterised as MtWD40-1 (ref). **C.** The wild-type *Zt1*/VfWD40-1 allele from NV648 with normal flower pigmentation. Primer pairs F1/R1 and F2/R1 amplify 1032bp and 582bp fragments respectively. Grey boxes show the locations of the 4 WD40 repeats. **D.** The recessive *zt1*/VfWD40-1 allele from white-flowered parent NV643 amplifies only the 582bp F2/R1 fragment in common with the wild-type allele and genome walking from the conserved 3' end shows that it contains only one WD40 domain (grey box) and strong homologies in the 5' flanking sequence to *Pisum sativum* adenylate isopentenyltransferase (PsITP1,Genbank Sequence ID AB194606.1; wavy pattern fill) and VfENOD12 (AJ277288.1; checkerboard pattern fill).
